# Regulation of PIN-FORMED Protein Degradation

**DOI:** 10.3390/ijms24010843

**Published:** 2023-01-03

**Authors:** Liuqin Zhang, Yifan Guo, Yujie Zhang, Yuxin Li, Yan Pei, Mi Zhang

**Affiliations:** 1 Biotechnology Research Center, Southwest University, No. 2 Tiansheng Road, Beibei, Chongqing 400715, China; 2 Chongqing Key Laboratory of Plant Resource Conservation and Germplasm Innovation, Southwest University, Chongqing 400715, China

**Keywords:** PIN-FORMED, degradation, vacuolar sorting, ubiquitin modification

## Abstract

Auxin action largely depends on the establishment of auxin concentration gradient within plant organs, where PIN-formed (PIN) auxin transporter-mediated directional auxin movement plays an important role. Accumulating studies have revealed the need of polar plasma membrane (PM) localization of PIN proteins as well as regulation of PIN polarity in response to developmental cues and environmental stimuli, amongst which a typical example is regulation of PIN phosphorylation by AGCVIII protein kinases and type A regulatory subunits of PP2A phosphatases. Recent findings, however, highlight the importance of PIN degradation in reestablishing auxin gradient. Although the underlying mechanism is poorly understood, these findings provide a novel aspect to broaden the current knowledge on regulation of polar auxin transport. In this review, we summarize the current understanding on controlling PIN degradation by endosome-mediated vacuolar targeting, autophagy, ubiquitin modification and the related E3 ubiquitin ligases, cytoskeletons, plant hormones, environmental stimuli, and other regulators, and discuss the possible mechanisms according to recent studies.

## 1. Introduction

PIN-formed (PIN) proteins are auxin efflux carriers that are extremely important for auxin-triggered organogenesis in plants. Early in 1998, it was uncovered that polar plasma-membrane localization behaviors of PIN proteins mediate polar auxin transport during inflorescence development and root gravitropism of *Arabidopsis thaliana* [1,2]. In total, eight members the PIN family are found in Arabidopsis. All of them typically have a central hydrophilic loop between two hydrophobic regions of five transmembrane domains each. Based on length of the central hydrophilic loop, PINs are generally divided into two groups: long-type PINs (PIN1–PIN4 and PIN7) that localize at the PM and regulate intercellular auxin transport as auxin efflux facilitators [3]; short-type PINs (PIN5 and PIN8) that localize at the endoplasmic reticulum (ER) and contribute to homeostasis [4,5,6]. PIN6, processing a middle length of the hydrophilic loop between long- and short-type PINs, shows dual localizations at both the PM and ER depending on its phosphorylation states and expression levels [7,8]. Polarity of PM-located PINs remodels auxin efflux behavior then controls directional cell-to-cell auxin transport in tissues to establish auxin gradient for the following activation of auxin signaling.

PINs, like other PM-localized transmembrane proteins, are distributed along with their resident vesicle bodies through the intracellular trafficking system. Establishment of PIN polarity involves delivery of new synthesized proteins to the PM by exocytosis and removal from the PM by endocytosis [9]. Once processes are disturbed among sorting, trafficking, and turnover (synthesis and degradation) of membrane proteins, the PIN abundance and polarity are affected. The intracellular trafficking of PINs most depends on the action of guanine nucleotide exchange factors for ADP ribosylation factors (ARF GEFs), including GNOM and GNOM-like1 (GNL1), and BFA-visualized endocytic trafficking defective (BENs) [10,11,12,13]. PIN phosphorylation regulated by AGCVIII protein kinases, such as PINOID, and by type A regulatory subunits of PP2A phosphatases, for example PP2AA1/RCN1, is a well-studied mechanism for repolarization of PIN localization [14,15]. Recent work highlights roles of PIN degradation in modulation of polar auxin transport, particularly regarding PIN2 in gravistimulated roots [16,17], providing a new aspect to understand the regulation of PIN-mediated polar auxin transport. This review does not attempt to cover all regulations on every process of PIN intracellular trafficking, but rather focuses on those involving the protein degradation of PINs in developmental contexts and the related ubiquitin modification, which have been recently revealed. Moreover, some other regulators, such as plant hormones and environmental stimuli, will also be discussed.

## 2. PIN Degradation

Turnover of proteins is crucial to maintain their normal function and to allow their levels to change quickly in response to stimuli in organisms. Membrane proteins, including PINs, are normally targeted to the vacuole for degradation [9]. Dark treatment makes PINs more stable in the vacuole and thus the vacuole-like accumulation of GFP-tagged PINs is visualized [18]. The vacuole-targeted PIN proteins can also be observed by treatment with concanamycin A (ConcA) [18], a vacuolar H^+^-ATPase inhibitor that neutralizes vacuolar pH and thus blocks trafficking to the vacuole [19]. Wortmannin, a PI3K and PI4K inhibitor [20], displays a similar inhibitory role in vacuolar targeting process of certain PINs in some special scenarios by forming different aggregations called WM compartments, also suggesting dependence of PIN vacuolar trafficking on PI3K activity [18]. In contrast, trafficking and endocytosis inhibitor 1/TENin1 (TE1) reversibly inhibits retrograde trafficking of membrane cargos (e.g., PIN2-GFP) from the multivesicular body (MVB) to the trans-Golgi network (TGN) and thus promotes vacuolar targeting, whilst displays an inhibitory effect on endocytic recycling [21].

To date, PIN2 relocalization in gravitropic response is the clearly documented scenario involving PIN regulation by protein degradation. PIN2 localizes at the PM of root cortical and epidermal cell files to establish a lateral auxin gradient in the root tip [2,22]. During gravistimulation, PIN2 distribution displays differential between the upper and the lower sides of roots to establish asymmetric distribution of auxin [16], which process is largely dependent on post-transcriptional mechanism [17]. The reduction of PIN2 signal at the upper side is due to the enhanced vacuolar targeting of PIN2, as visualized by dark treatment [18]. The interference with asymmetric distribution of PIN2 by treatment of MG132, a 26S proteasome inhibitor, indicates that gravi-induced proteolysis of PIN2 depends on proteasome-dependent manner [16]. Although the involvement of the 26S proteasome in PIN2 vacuole targeting is also reported in another study [23], the accurate function of proteasomes, commonly known as degradation machineries for soluble and cytosolic proteins, in PIN proteolysis is still in mystery to date. Nevertheless, the report that MG132 treatment relieved the inhibition of histone deacetylase inhibitors on PIN1-GFP accumulation in roots, where PIN1 transcription was unchanged, suggests a possible link between epigenetic regulation and 26S proteasome-mediated PIN degradation [24].

Autophagy is an evolutionarily conserved intracellular degradation pathway, which is characterized by the formation of double-membrane autophagosomes, for eukaryotic cells in response to the dynamic change of nutrients and environmental stress. Recent studies suggest that this system, as integration of multiple signaling, including TOR (target of rapamycin), glucose, and hormones, also participated in selective degradation of PIN proteins [25,26]. Tens of conserved autophagy-related genes (ATGs) have been identified as core players of the autophagic machinery in yeast, animals, and plants [27]. Loss of function of ATG5 and ATG7, both responsible for phagophore formation, alters PIN1-GFP accumulation in roots and the variation tendency of the intensity under glucose induction [28], implying that PIN protein can be degraded through autophagic pathway. Target of rapamycin (TOR), a phosphatidylinositol kinase-related kinase (PIKK) family member, acts as a core integrator of nutrient and energy signaling, and further negatively regulates autophagic activation in plants [29]. The latest study revealed that PIN2 is a downstream target of glucose-TOR signaling, by which glucose-activated TOR phosphorylates and stabilizes PIN2 and therefore influences root cell elongation [25].

## 3. Ubiquitin Modification

Ubiquitin modification acts as a sorting signal for integral membrane proteins [30]. Numerous studies have indicated that ubiquitination of PIN-formed proteins triggers their protein degradation in the vacuole. In Arabidopsis roots, vacuolar targeting and proteolytic turnover of PIN2 are associated with PIN2 ubiquitination status [16,31], which undergoes a continuous enhancement in initially several hours after gravistimulation [32]. Although PIN2 stability and ubiquitination level depend on 26S proteasome [16], through which Lysine-48 (K-48) linked polyubiquitin chains of soluble proteins act as the canonical signal for degradation [30], K-63 ubiquitination that is required for the vacuole targeting of membrane proteins is detected as the major form of PIN2 polyubiquitination [31]. As membrane proteins cannot directly access to the proteasome, the involvement of proteasome activity may facilitate the trafficking process of PINs to the vacuole [16]. In mammalian and yeast systems, the ubiquitinated membrane proteins, in addition to direct traffic of misfolded proteins from the Golgi to endosome or subjection to ER-associated degradation (ERAD), are generally endocytosed and targeted to the lysosome/vacuole for degradation [30]. This idea is supported by work studying PIN2 in Arabidopsis. Mutation of multiple lysines, the potential ubiquitination sites residing in the central hydrophilic loop of PIN2, to arginines (K to R) largely impairs the K-63 linked polyubiquitination and root gravitropism [31]. The lysine substitutions of PIN2 result in stronger PM localization and ectopic accumulation, which is due to deficiency in endocytic sorting and vacuolar degradation [31]. In contrast, constitutive ubiquitination modification of PIN2, by fusion with ubiquitin, displays enhanced endocytosis and proteolytic degradation [31]. PIN regulation by ubiquitination also participates in cell-type-dependent vacuolar degradation of PIN2 in tricho- and atrichoblasts, where either the lysine substitution or constitutive ubiquitination of PIN2 abolishes the differential PM abundance [33].

In contrast to ubiquitination, deubiquitylation has also been implicated in PIN degradation. Associated molecule with the SH3 domain of STAM3 (AMSH3), a major Arabidopsis deubiquitinating enzyme (DUB) that hydrolyzes K48- and K63-linked ubiquitin chains but is independent of the 26S proteasome, regulates ubiquitin-mediated endocytic degradation [34,35]. Mutation in AMSH3 causes defect in vacuole formation and accumulation ubiquitinated membrane proteins, and thus results in disability of PIN2 vacuolar degradation [34]. Analogous phenotypes can be found in the mutant of *amsh1* and the plant overexpressing dominantly negative form of VPS2.1, the ESCRT-III (discussed below) subunit that interacts with AMSH1 or AMSH3 [35,36]. In addition, enhanced accumulation of autophagosomes in *amsh3*, darkness-treated *amsh1*, and the dominant mutant of VPS2.1 [34,36] also suggests the possible association between PIN degradation, ubiquitin modification, and autophagy.

## 4. Ubiquitin E3 Ligases

Currently, several ubiquitin E3 ligases, which recognize and transfer ubiquitin to the substrate and thus determine substrate specificity [37], have been revealed to control PIN protein stability. Ring domain ligase1 (RGLG1) and RGLG2 act redundantly in K-63 polyubiquitination and auxin-regulated development events [38]. Reduced accumulation of PIN1-GFP, as well as PIN2-GFP, in *rglg1rglg2* roots indicates that PIN1 and PIN2 are unlikely to be direct targets of RGLGs for ubiquitination-mediated degradation, although physical interaction between RGLG2 and PIN1 was detected in the yeast-two-hybrid assay [38]. Interestingly, a following study reports increased PIN2 protein levels and reduced PIN2 K-63 ubiquitination in *rglg1 rglg2*, suggesting a definite role of RGLG1 and RGLG2 in controlling PIN stability through K-63 ubiquitination [31]. Constitutive photomorphogenic1 (COP1), a RING E3 ubiquitin ligase playing a core role in photomorphogenesis and skotomorphogenesis, regulates PIN localization in different ways. In shoots, COP1 delivers light signal to roots and influences root growth, through transcriptional reduction of PIN1 in hypocotyls, and thus, modulation of PIN1-mediated basipetal auxin transport [39]. In roots, COP1, after perception of auxin transported from shoots, participates in dark induced reduction of PM-located PIN1-GFP and PIN2-GFP, as well as vacuolar accumulation of PIN2-GFP in different posttranscriptional mechanisms, because inhibition of proteasome activity by lactacystin blocked PIN2 vacuolar targeting, whereas not the intracellular distribution of PIN1-GFP [39]. Moreover, defect of PIN2 redistribution and root reorientation during gravistimulation suggest participation of COP1 in PIN2 stability in gravitropism [39]. It remains unknown how COP1 regulates PIN localization and whether COP1 directly ubiquitinates PINs for degradation, but effect of HY5, a downstream substrate of COP1-dependent light signaling on PIN2 localization [23] may give a hint of involvement of COP1-HY5 module in PIN2 vacuolar targeting.

## 5. PI3K Complexes

PI3K activity plays pivotal roles in various cellular processes, such as endocytic trafficking and autophagy, by production of phosphatidylinositol 3-phosphate (PI3P), which provides a binding site for proteins with certain lipid binding domains, including FYVE and PX domains [40]. Two main PI3K complexes are implicated in regulation of PIN proteins. The PI3K complex I, composed of vacuolar protein sorting34 (VPS34, a membrane of Class III phosphoinositide 3-kinase family), VPS15, VPS30/ATG6/Beclin1, ATG14 and ATG38, is essential for autophagosome biogenesis [41,42], while the other PI3K complex II, consisting of VPS34, VPS15, VPS30/ATG6/Beclin1, and VPS38, is generally required for ESCRT-mediated MVB formation, autophagosome-lysosome fusion, and the function of the retromer complex, which controls endosome to Golgi retrograde trafficking [40]. Due to diverse functions of PI3K complexes and additional effect by PI3K inhibitors on phagophore formation [43], PIN degradation by endosomal sorting and autophagy is ambiguous based on current knowledge. Moreover, recent studies have revealed that both autophagosomes and PVCs act together in regulating protein degradation in plants [44].

VPS38, as a component of PI3K complex II, has a profound role in modulating late endosome/MVB morphology [45,46]. Mutation of VPS38 resulted in abnormal cytoplasmic distributions of GFP-PIN1, -PIN2, and -PIN3 [45,46,47]. The association of VPS38 with VPS30 and VPS34, another two components of PI3K complex II, and the colocalization of VPS38 and retromer VPS29 [46,47] indicate that the regulatory role of VPS38 on PIN localization is more dependent on PVC sorting, although *vps38* mutants showed defects in autophagy [45,46].

Recent studies revealed that phosphatidylinositol-4-phosphate (PI4P), product of phosphatidylinositol 4-kinase (PI4K), is required for autophagosome formation in Arabidopsis [48]. The finding that a double mutant in PI4K β1 and β2 isoforms exhibited no PIN2-GFP signal in vacuoles under darkness suggests the regulatory role of PI4K in PIN vacuole targeting [49]. Despite the altered F-actin organization in *pi4kβ1β2* implies a possible mediation of F-actin [49], the underlying mechanism remains future elaboration.

## 6. SNAREs

Soluble N-ethylmaleimide sensitive factor attachment protein receptors (SNAREs) mediate fusion between vesicular and target membranes. Usually, some of these transmembrane proteins reside on vesicular membranes in a single chain, while the others reside on target membranes in a three-protein complex [50,51]. The assembly of SNAREs residing on both membranes to a stable four-helix bundle leads to membrane fusion. VAM3/SYP22 is a SNARE that localizes to both the MVB and the tonoplast. Since *vam3* mutant displayed depolarization of PIN1-GFP in leaves and vacuolar aggregation of PIN1-GFP was surrounded by mRFP-VAM3 labeled tonoplast under dark treatment, it is suggested that VAM3-mediated vacuolar trafficking is required for maintenance of PIN polarity [52]. In addition, mutation in both SYP42 and SYP43 (*syp42 syp43*), two SYP4 group members of TGN-localized SNARE [51], led to defect of vacuolar accumulation of PIN2-GFP in the darkness [53], also providing an indication of involvement of SYP4 in PIN vacuolar transport. Nonetheless, the regulatory effect of SNARE SYP4 is indirect and could be attributed to the influence on trafficking between the TGN and MVBs [53]. Interestingly, VAM3 did affect localization behaviors of PIN1-GFP and PIN2-GFP in roots [52]. How these SNARE complexes determine specificity of membrane fusion is still unclear. Another TGN-located SNARE VTI12 may also participate in the regulation of PIN vacuolar degradation as its interaction with EpsinR2, which also interacts with adaptor protein3 (AP3) complex [54] that regulates conversion of storage vacuoles into lytic ones [55]. Disturbance of AP3 function by mutations in AP3-ß3 or -δ subunits, as well as by overexpression of dominant negative AP3-μ3 subunit, resulted in aberrantly accumulated cargos, including PIN1, in vacuole-like compartments [55,56]. Phe-165 of PIN1 is required for the binding to AP3-μ3 subunits and PIN1 vacuole targeting [57]. Interestingly, AP3-ß3-GFP labeled endomembrane compartments were shown to be insensitive to BFA and wortmannin treatment [55], implying an uncharacterized trafficking pathway not through TGN, Golgi apparatus and MVBs.

## 7. The Retrograde Systems

The retromer is a conserved protein complex that regulates vesicular transport of transmembrane proteins from endosomes to the trans-Golgi network (TGN) and to the plasma membrane [58]. It consists of a dimer of sorting nexins (SNXs), which contains PX domains binding to phosphatidylinositol 3-phosphate (PI3P) produced by VPS34 for membrane recruitment, and a trimer of VPS26, VPS29, and VPS35, which binds endocytic receptors for cargo selection [59]. In Arabidopsis, the retromer protein VPS29, as well as the physical interactors VPS35 and VPS26, colocalize with SNX1 at the MVB and are required for MVB morphology [18,60,61]. The complex controls endocytic recycling of specific cargos, such as PIN1 and PIN2, because loss of function of VPS29 just resulted in intracellular accumulation of PIN1-GFP and PIN2-GFP, but not AUX1-YFP and GFP-PIP2a, in SNX1-labled compartments [61]. The improperly recycled PIN proteins in *vps29* mutant were then destinated for degradation [18,61]. Similar alteration of PINs is also reported in *snx1* mutants [18,62]. A posttranslational regulation is suggested in such context based on the finding that the decreased PIN2 protein levels in *snx1* mutants are independent of gene transcription [18]. Recent work reveals that SNX1 forms a complex both with phosphatidylinositol 3-phosphate 5-kinase (FAB1) and its product phosphatidylinositol 3,5-bisphosphate (PtdIns(3,5)P_2_, which is required for the association of SNX1 with the endosome membrane) to facilitate maturation of SNX1 endosomes, and hence to impair PIN2 trafficking [63]. In addition, BLOC1 complex that interacts with SNX1 on sorting endosomes, mediates PIN vacuolar sorting for degradation via the retromer pathway [64]. Further work suggests that plants employ this regulation of PINs, particularly PIN2, by the retromer to response to environmental stimuli, including darkness, gravity and high temperature [18,65]. Moreover, a messenger molecule inositol 1,4,5-trisphosphate (Ins(1,4,5)P_3_) is able to enhance endocytosis, but not exocytosis, of SNXs to reduce their localization at the PM and thus to suppress PIN2 degradation in vacuoles [66].

Like the retromer, the Golgi-associated retrograde protein (GARP) complex is also part of evolutionarily conserved cellular inter-compartmental transport systems. A tetramer of the GARP complex consists of VPS51, VPS52, VPS53, and VPS54 subunits, and is required for retrograde trafficking from both the early and late endosomes to the Golgi [67]. Proper PIN1 protein distribution in leaf margins is dependent on function of UNHINGED (UNH), an Arabidopsis VPS51 homolog, which presumably promotes PIN1 targeting to the vacuole [68], suggesting a critical role of the GARP complex in regulating PIN vacuolar degradation.

## 8. The ESCRT Pathway

Sorting into the intralumenal vesicles of endosomes is a common step for membrane protein degradation in vacuoles. This process is controlled by the ESCRT (endosomal sorting complex required for transport), which is defined as a ubiquitin-dependent protein sorting pathway consisting of five distinct complexes, termed ESCRT-0, ESCRT-I, ESCRT-II and ESCRT-III, and vacuolar protein sorting4 (VPS4) [69,70]. These complexes are sequentially required to interact with ubiquitinated membrane proteins and to drive their internalization into the intraluminal vesicles of MVBs. Therefore, the components of such complexes are involved in PIN degradation in vacuoles.

TOM (target of MYB) 1-likes (TOLs) have VHS (Vps27, Hrs, and STAM) and GAT (GGAs and TOM) domains similar to components of the ESCRT-0, which is essential for recruitment of the ESCRT-1 for initiating MVB-dependent cargo sorting. They function redundantly in recognizing ubiquitinated PINs and then regulating the endocytic sorting to vacuoles for protein degradation [71]. Another ESCRT-0 subunit identified in plants is FYVE domain protein required for endosomal sorting1 (FREE1), which binds to PI3P, ubiquitin, and specifically interacts with VPS23A and VPS23B, subunits of the ESCRT-I complex, via PTAP-like tetrapeptide motifs [72]. Mutation in FREE1 led to defects in MVB formation and mislocalization of vacuole-accumulated endocytosed PIN2 to the tonoplast [72]. To some extent, the ESCRT-0 together with ubiquitin modification determines specificity of vacuolar targeting of membrane proteins, because multiple mutation of TOLs caused defect in forming WM compartments of PIN2-VENUS while not in that of FM4-64 labeled endomembrane [71]. It is noteworthy that FREE1 does not merely regulate protein sorting to the vacuole but also autophagic degradation because of association between FREE1, SH3P2 (a unique plant autophagy regulator), and the PI3K complex I subunit ATG6 [73]. ELC is an Arabidopsis homolog of VPS23 displaying the binding activity to ubiquitin. It forms the ESCRT-I complex with homologs of VPS37 and VPS28 [74]. VPS28A and VPS28B are redundant components of the ESCRT-I complex involved in Arabidopsis embryonic development. In embryos of *vps28avps28b* double mutant, PIN1-GFP displayed broadened expression profile, reduced polarity, and aberrant vacuole-like structures [75]. Aberrant localization and polarity of PIN1-GFP and PIN2-GFP are also reported in the knockout mutant of VPS36, the ESCRT-II subunit that can bind ubiquitin and interact with the other ESCRT-II components VPS22 and VPS25 [76]. Although no direct evidence supports the involvement of the ESCRT-III complex VPS2.1 and VPS24.1 in PIN localization, the regulatory role is undoubted due to their interaction with AMSH3, a deubiquitinating enzyme that controls intracellular trafficking and vacuole targeting of PIN2-GFP [34,35]. Moreover, enhanced stability was indeed observed in seedlings overexpressing VPS2.1-GFP that disturbs the ESCRT-III function [36]. Moreover, the defective autophagic degradation in such seedlings [36] indicates the function of the ESCRT in targeting autophagosomes to the vacuole. In addition, two ESCRT-III accessory proteins charged multivesicular body protein/chromatin modifying protein1A (CHMP1A) and CHMP1B are found to mediate MVB sorting pathway of PIN1 and PIN2 [77]. CHMP1A is also capable to interact with SKD1 and LIP5, two VPS4 complex subunits [77]. SKD1, the only ATPase in the core ESCRT machinery, is required for disassembly and recycling of the ESCRT-III accessory back to the cytoplasm and the continuous release of intraluminal vesicles into the endosomal lumen. Loss of function of LIP5, a positive modulator of SKD1 activity, prevented PIN2-GFP and PIN3-GFP targeting to the vacuolar lumen for degradation, indicating a necessary role of LIP5 for intraluminal vesicles formation, which likely requires interaction of LIP5 and the ESCRT-III accessory subunit ISTL1 [78].

## 9. Cytoskeletons

Both microtubule and actin cytoskeletons that are responsible for intracellular vesicular trafficking have been implicated in regulating PIN targeting to the vacuole. Especially for filamentous actin (F-actin), auxin transporter inhibitors, such as 2,3,5-triiodobenzoic acid (TIBA) and 2-(1-pyrenoyl) benzoic acid (PBA), also serve as actin stabilizers and repress subcellular motility in plant cells [79]. Nevertheless, the inhibition of PIN2 vacuolar targeting by latrunculin B (LatB)-induced actin depolymerization [18] implies a different effect of F-actin that the stabilization may promote vesicular trafficking to the vacuole. This finding is supported by appearance of reduced PM abundance and increased intracellular aggregation of PIN2-GFP in *act7*, the loss-of-function mutant of actin subunit ACT7 with excessive actin bundling [80]. The contradiction is possibly due to inconsistent effects of various inhibitors on consequently F-actin dynamics or different roles of the actin cytoskeleton in subcellular trafficking in different scenarios. Comparing to the F-actin, few studies report the role of microtubules in PIN vacuolar sorting. Given that a microtubule-associated protein cytoplasmic linker associated protein1 (CLASP1) positively regulates microtubule depolymerization, the interaction with retromer component SNX1 indicates involvement of microtubules in PIN2 retrograde trafficking from endosomes [81]. Moreover, Hirano et al. (2015) recently suggested that the interplay between microtubules and FAB1 endosomes, through which oryzalin-caused microtubule depolymerization disturbs maturation of those endosomes, promotes gravistimulation-dependent PIN2 degradation [63]. However, further exploration of how microtubule cytoskeleton as well as its dynamics participates in maintenance of endosome structures is required.

## 10. Plant Hormones

Plant hormones are critical regulators for PIN degradation. The most studied is auxin as shown in the experiment that treatment with either naphthylene-1-acetic acid (NAA), an artificially synthesized auxin, or 2,3,5-triiodobenzoic acid (TIBA), a polar auxin transport inhibitor, reduced PIN2 protein levels with unaffected transcription levels [16,82]. The scenario of NAA-induced PIN2 degradation is further specified to PIN2 recovery decrease at the lower side of gravistimulated roots after the initial increase of PIN2 level. Meanwhile, at the upper side of the bending roots where PIN2 levels initially decrease, auxin depletion also promotes PIN2 vacuolar targeting for degradation [17]. Both scenarios of PIN2 turnover regulation involve SCF^TIR1/AFB^-based auxin signaling pathway and suggest optimal auxin levels for the stabilization of PIN2 proteins [17,82]. Nonetheless, no auxin signaling components have been revealed with differential expression between the upper and lower part of root during gravistimulation, also indicating that the initial PIN2 degradation there might not be triggered by the lack of auxin. Ubiquitin modification, at least that of PIN2, might prompt new thinking for insight into regulatory mechanism of PIN degradation. It is also worth noting that auxin has an inhibitory effect on PIN2 endocytosis and thus promotes its PM localization [83], which is achieved by repression of membrane sterol synthesis through SCF^TIR1/AFB^-dependent auxin signaling [84]. As the endocytosis assay largely depends on BFA treatment, this view is argued by later evidence that auxin cannot inhibit PIN2 endocytosis and the BFA-based approach is inappropriate [85].

Recent studies reveal that gibberellic acid (GA) displays dual regulation of PIN trafficking—promoting PIN vacuolar targeting for degradation at low GA levels, or promoting the recycling to the PM at high GA levels [86,87]. The retromer complex and MT cytoskeleton are required in this regulatory process under GA signaling mediated by DELLA proteins, which is a post-transcriptional regulation possibly through the interaction between DELLAs and Prefoldins, regulators for tubulin folding [88].

Cytokinin, frequently having crosstalk with auxin, acts as a PIN regulator at both transcriptional and post-translational levels. Take PIN1 for example, B-type ARR cytokinin response factors bind directly to the responsive elements, including PIN1 cytokinin response element (PCRE1), in PIN1 promoter to upregulate PIN1 expression in inflorescences [89]. However, in terms of regulation of PIN degradation, cytokinin, during development of lateral root primordium, targets PIN1 to the vacuole for degradation through modulation of the endocytic trafficking. Interference of the cytokinin effect by latrunculin B-caused actin depolymerization, not by that of oryzalin-induced microtubule depolymerization, suggests the mediation of the actin cytoskeleton in the process [90]. In addition, cytokinin perception exclusively by AHK4 receptor and signaling transduction by some B-type ARR is also required [90].

Recent work has revealed the inhibitory role of ethylene in regulation of PIN2 vacuolar targeting via stabilizing shade avoidance4 (SAV4) protein [91]. SAV4 was first identified as a regulator of auxin efflux carrier ABCB1 that inhibits auxin transport activity [92]. In root epidermal cells, SAV4 shares similar polar localization at the plasma membrane with PIN2. Due to the direct interaction with PIN2 hydrophilic loop, the stabilization of SAV4 by ethylene impairs PIN2 vacuolar trafficking possibly by recruiting PIN2 to the PM. However, it is noteworthy that PIN2 dynamic was enhanced in *sav4* mutant, not only the trafficking to the PM but also that to the vacuole [91]. The effect of SAV4 on MT organization still needs to be considered since SAV4 also interacts with microtubule-associated protein ABS6 and promotes MT severing [93].

In terms of jasmonic acid (JA) and abscisic acid (ABA), although it is reported that they are able to reduce PM abundance and protein level of PIN2 [94,95], the analysis by photoconvertible protein Dendra2 suggests that the effect by these two hormones is possibly attributed to the transcriptional downregulation of PIN2 [95]. Moreover, the latest study reveals that, under stress condition, ABA-induced expression of a stress-responsive asparagine rich protein (NRP) in turn promotes PIN2 vacuolar degradation to inhibit root elongation [96], suggesting a post-translational regulation of ABA on PIN proteins.

## 11. Environmental Stimuli

Gravity is the most mentioned trigger of PIN degradation, which has been discussed regarding PIN2 redistribution in root gravitropism. The perception of the gravity vector is tightly associated with statoliths, dense starch-filled organelles which settle to the vicinity of the plasma membrane of the columella of the root cap and the endodermal cells of aerial tissue under gravistimulation [97]. Although the physical attachment of amyloplasts to the cytoskeleton and activation of calcium release from the endoplasmic reticulum by statoliths suggest the signal transduction during gravitropic response and reorient plant growth via auxin [98], the detailed link of gravity perception to the establishment of auxin gradient remains unidentified to date. In addition, light negatively regulates PIN2 vacuolar targeting in HY5-dependent pathway, but the mechanism is still large in the shadow and 26S proteasome and COP9 signalosome, which directly modulates ubiquitin E3 ligase function for26 proteasome-mediated degradation, are involved in this process [23]. Temperature may be another factor influencing PIN turnover, as evidenced by sHSP22, an endoplasmic reticulum (ER) small heat shock protein, of which gene transcription is greatly induced by auxin with the involvement of ABI1, a key negative regulator of ABA signaling [99]. In roots, sHSP2 negatively regulates accumulation of PINs (such as PIN1, PIN3 and PIN7) in a posttranscriptional manner, possibly because of promotive effect of sHSP22 on PIN internalization [99]. Phosphorus (Pi) deficiency is reported to inhibit PIN2 vacuolar degradation through PHOSPHOLIPASE D (PLD2)-mediated phosphatidic acid (PA) [100]. As in the condition of Pi starvation, transcription of PLDs, as represented by PLDζ2, is strongly induced, and further promotes production of PA. PA then enhances accumulation of the retromer at the PM by direct binding with SNX1, and thus, results in reduced PIN2 endocytosis, which consequently suppresses PIN2 vacuolar targeting for degradation.

## 12. Other Regulators

In addition, some other genes are reported to regulate PIN degradation. MODULATOR OF PIN2 (MOP2) and MOP3, which are identified by a genetic screen, control PIN stability in a nonredundant manner as mutation of either reduced PIN protein levels [101], however, the underlying mechanism of these newly characterized PIN regulators is still unknown. During establishment of procambial cells in cotyledons, expression and localization are under the regulation of vasculature complexity and connectivity (VCC), a plant-specific transmembrane protein which expression is induced by auxin. As VCC mutated, depolarization and vacuolar accumulation of PIN1-GFP were enhanced [102]. SSR1 is a mitochondrial protein that regulates the retrograde trafficking possibly through the influence on the retromer. Knock-out of SSR1 resulted in decrease of protein expression of canonical PINs in roots, especially for PIN2 that underwent rapid degradation [103]. *MAB1* encodes a mitochondrial E1ß subunit of the pyruvate dehydrogenase complex in the TCA cycle. The amounts of PIN1 and PIN2 at the PM were reduced in *mab1* in a transcriptional behavior, likely due to the reduction of PIN endocytic recycling and acceleration of PIN vacuolar sorting [104]. Loss-of-function and dominant-negative (DN) mutations in ROP3 (Rho-related GTPase of plants 3) also cause reduced protein levels of PIN1-GFP and PIN3-GFP, which was inhibited by MG132 treatment. In the meantime, vacuole-localized PIN1-GFP signal was observed in roots of these mutants treated with MG132, providing indication of vacuolar degradation [105]. The effects may be not limited to ROP3, but expanded to other ROPs, as PIN3-GFP was less affected in such context than PIN1-GFP [105], and dysfunction of ROP2 and ROP6, like that of ROP3, also reduced PIN abundance at the PM and induce intracellular aggregation of PIN-GFPs [106,107,108]. However, ROPs are more likely to be the regulator of PIN intracellular trafficking, rather than the switch to trigger PIN proteolysis, because, take ROP6 for example, the function of inhibiting PIN2 endocytosis and thus resulting in more PIN2 at the PM is unable to interpret the differential distribution of PIN2 in bending roots during gravistimulation. Similar action on PIN regulation can also be seen in the upstream players of ROP6 signaling pathway, including phosphatidylserine [109], transmembrane kinase1 (TMK1) [110], membrane associated kinase regulator2 (MAKR2) [110], SPIKE1 (SPK1) [108], as well as the downstream targets RIC1 and actin filaments [108].

## 13. Conclusions

**Figure 1:**
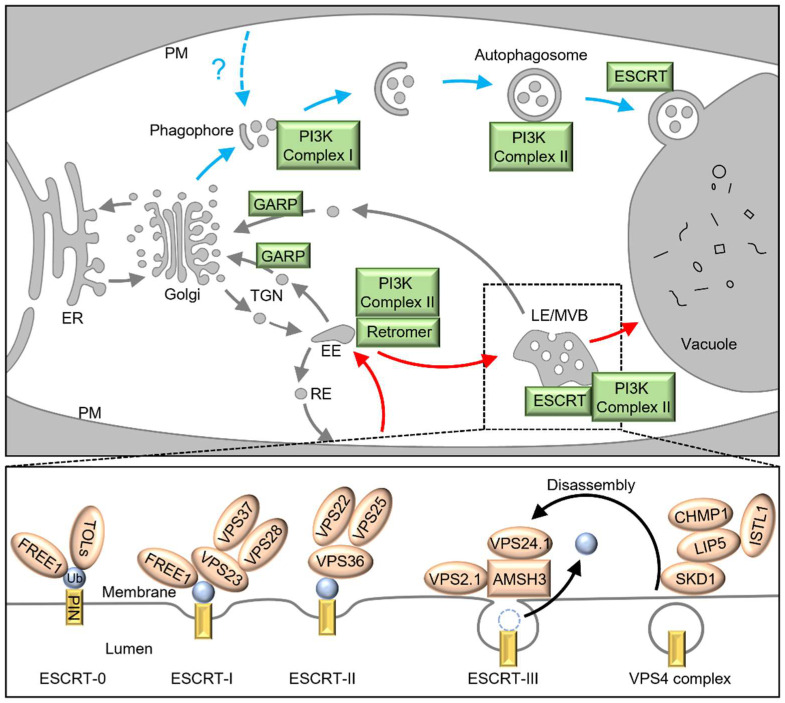
An illustration of PIN degradation in plants. Ubiquitinated PINs are proposed to be endocytosed from the PM for degradation via endosome-mediated vacuolar targeting (red arrows). The ESCRT mediates the sorting of PINs into the intraluminal vesicles of endosomes as highlighted in MVBs and the detailed process is shown in the lower panel. CHMP1 and ISTL1 are two subunits of the ESCRT-III, each influencing activity of SKD1 via interaction with LIP5. Recent work also suggests the involvement of autophagy in PIN degradation (blue arrows), although the explicit process is largely unknown and how PIN-residing membrane structures are integrated into the autophagic pathway remains in mystery. The ESCRT is also required for fusion of autophagosomes to the vacuole. ER, endoplasmic reticulum; TGN, trans-Golgi network; EE, early endosome; PM, plasma membrane; RE, recycling endosome; LE/MVB, late endosomes/multivesicular body.

The recent findings highlighted in this article, including vacuolar target, ubiquitin modification, vesicular trafficking, cytoskeletons, plant hormones, and environmental stimuli, expand the known regulation of PIN degradation. In addition, these newly identified mechanisms set the stage for a much broader understanding of endomembrane trafficking in plant cells. Many gaps remain in understanding the processes that drive PIN proteolysis. Despite much clearer evidence for many players involved in vesicular trafficking and fusion (Figure 1 and Table 1), we still know little about how environmental signals or developmental cues are transduced to plant cells and further trigger PIN degradation. Moreover, the direct regulator for PIN proteolysis is still unknown. More solid evidence is required to identify ubiquitin E3 ligases that recognize PIN proteins as direct substrates, as well as upstream signaling that activates the ubiquitin modification, although part of RGLG2 has been reported to interact with the cytoplasmic domain of PIN1 in vitro [38]. It is still questionable where PINs are ubiquitinated, on the PM or the other endomembrane structures, if ubiquitin modification acts as the trigger of PIN degradation. The PM localization of TOL6 and defective vacuolar targeting of PIN2 in a quintuple mutant of TOLs [71], at least, indicates ubiquitin modification of PINs at the PM is sufficient for the degradation. As in some cases, different from those are implicated in intracellular trafficking, the regulation of vacuolar targeting is exclusive to PIN proteins, even an individual PIN member, whether ubiquitin modification determinates the specificity or what is the determinator are subjected to further identification. In addition, a noteworthy point is which pathways, including conventional endosome-mediated degradation, autophagy, and the 26S proteasome, account for degradation of PIN proteins. It is probably scenario-dependent, and interplay of these pathways should be concerned. Although several studies report the inhibition of PIN degradation by MG132, no evidence supports K-48 polyubiquitination of PINs, which target the substrate to the proteasome for degradation. There is no doubt that root gravitropism, the extensively studied scenario for PIN2 degradation, will be the model system to address these questions. However, more elaborative work, especially the temporal information of core components within the regulatory pathway in the upper and lower sides of gravi-stimulated roots, is required to broaden our understanding of regulation of PIN degradation. Moreover, cotton fibers, where a PIN homolog (GhPIN3a) is preferentially expressed and undergoes cell-specific degradation during cell initiation [111], provide another cell model for elaboration of the regulation.

## Figures and Tables

**Table 1:** Intracellular components involved in PIN vacuolar sorting.

Gene	Complex	Process	Reference
TOLs	ESCRT-0	Recognition of ubiquitinated PIN2, PIN2 vacuolar targeting, root gravitropism, development of cotyledons, inflorescences and meristems	Korbei et al., 2013 [71]
FREE1	ESCRT-0	Recognition of ubiquitinated PIN2, PIN2 targeting to the tonoplast	Gao et al., 2014 [72]; Gao et al., 2015 [73]
VPS28A, VPS28B	ESCRT-Ⅰ	PIN1 polarity, vacuole formation, embryogenesis	Liu et al., 2020 [75]
VPS23/ELC	ESCRT-Ⅰ	Interaction with FREE1, interaction with ubiquitin, interaction with VPS28 and VPS37, cytokinesis, embryogenesis	Gao et al., 2014 [72]; Spitzer et al., 2006 [74]
VPS37	ESCRT-Ⅰ	interaction with VPS28 and VPS23	Spitzer et al., 2006 [74]
VPS36	ESCRT-Ⅱ	Polarity of PIN1/2, interaction with ubiquitin, VPS22 and VPS25, embryogenesis	Wang et al., 2017 [76]
VPS22	ESCRT-Ⅱ	Interaction with VPS25 and VPS36	Wang et al., 2017 [76]
VPS25	ESCRT-Ⅱ	Interaction with VPS22 and VPS36	Wang et al., 2017 [76]
VPS2.1	ESCRT-III	Interaction with AMSH3, intracellular trafficking, PIN vacuolar targeting	Isono et al., 2010 [34]; Katsiarimpa et al., 2014 [35]
VPS24.1	ESCRT-III	Interaction with AMSH3, intracellular trafficking, PIN vacuolar targeting	Isono et al., 2010 [34]; Katsiarimpa et al., 2014 [35]
CHMP1A	ESCRT-III	MVB sorting of PIN1,2, embryogenesis	Spitzer et al., 2009 [77]; Buono et al., 2016 [78]
CHMP1B	ESCRT-III	MVB sorting of PIN1,2, embryogenesis	Spitzer et al., 2009 [77]
ISTL1	ESCRT-III	Interaction with LIP5 to inhibit SKD1 activity	Buono et al., 2016 [78]
LIP5	VPS4 complex	Interaction with CHMP1A and ISTL1, inhibition of SKD1 activity, cargo (PIN2,3) sequestration into ILVs, root gravitropism	Spitzer et al., 2009 [77]; Buono et al., 2016 [78]
SKD1	VPS4 complex	interaction with CHMP1A, recycling of the ESCRT-III	Spitzer et al., 2009 [77]; Buono et al., 2016 [78]
VPS29	Retromer	Vesicular trafficking from endosomes to the TGN and to PM, MVB morphology, PIN2 vacuolar sorting and stability, root responses to light, gravity and high temperature	Jaillais et al., 2007 [61]; Kleine-Vehn et al., 2008 [18]; Yuan et al., 2018 [47].
SNX1	Retromer		Jaillais et al., 2007 [61]; Kleine-Vehn et al., 2008 [18]; Hanzawa et al., 2013 [65];
VPS35	Retromer		Seaman, 2012 [59].
VPS26	Retromer		Seaman, 2012 [59].
UNH	GRAP	PIN1 targeting to the vacuole	Pahari et al., 2014 [68].
VAM3/SYP22	SNARE	PIN1 polarity, PIN1 vacuolar trafficking, leaf development	Shirakawa et al., 2009 [52]
SYP42, SYP43	SNARE	PIN2 vacuolar transport	Sanderfoot, 2007 [51]; Uemura et al., 2012 [53]
VTI12	SNARE	PIN vacuolar transport mediated by EpsinR2 and AP3	Lee et al., 2007 [54]

## Data Availability

Not applicable.
